# The transcription factor CREM drives an inflammatory phenotype of T cells in oligoarticular juvenile idiopathic arthritis

**DOI:** 10.1186/s12969-018-0253-x

**Published:** 2018-06-20

**Authors:** Kim Ohl, Helge Nickel, Halima Moncrieffe, Patricia Klemm, Anja Scheufen, Dirk Föll, Viktor Wixler, Angela Schippers, Norbert Wagner, Lucy R. Wedderburn, Klaus Tenbrock

**Affiliations:** 1 Department of Pediatrics, Medical Faculty, RWTH Aachen, Pauwelsstr. 30, D-52074 Aachen, Germany; 2 Center for Autoimmune Genomics & Etiology, Cincinnati Children’s Hospital Medical Center, Cincinnati, OH USA; 3 Department of Pediatrics, University of Cincinnati, College of Medicine, Cincinnati, OH USA; 4 Department of Pediatric Rheumatology and Immunology, University Hospital Muenster, Muenster, Germany; 5 Institute of Virology, Westfaelische Wilhelms University, 48149 Muenster, Germany; 6 Arthritis Research UK Centre for Adolescent Rheumatology at UCL UCLH and GOSH, London, UK; 7 UCL GOS Institute of Child Health, University College London, London, UK; 8 NIHR- Great Ormond Street Hospital Biomedical Research Centre (BRC), London, UK

**Keywords:** CREM, JIA, Effector T cells

## Abstract

**Background:**

Inflammatory effector T cells trigger inflammation despite increased numbers of Treg cells in the synovial joint of patients suffering from juvenile idiopathic arthritis (JIA). The cAMP response element (CREM)α is known to play a major role in regulation of T cells in SLE, colitis, and EAE. However, its role in regulation of effector T cells within the inflammatory joint is unknown.

**Methods:**

CREM expression was analyzed in synovial fluid cells from oligoarticular JIA patients by flow cytometry. Peripheral blood mononuclear cells were incubated with synovial fluid and analyzed in the presence and absence of CREM using siRNA experiments for T cell phenotypes. To validate the role of CREM in vivo, ovalbumin-induced T cell dependent arthritis experiments were performed.

**Results:**

CREM is highly expressed in synovial fluid T cells and its expression can be induced by treating healthy control PBMCs with synovial fluid. Specifically, CREM is more abundant in CD161^+^ subsets, than CD161^−^ subsets, of T cells and contributes to cytokine expression by these cells. Finally, development of ovalbumin-induced experimental arthritis is ameliorated in mice with adoptively transferred CREM^−/−^ T cells.

**Conclusion:**

In conclusion, our study reveals that beyond its role in SLE T cells CREM also drives an inflammatory phenotype of T cells in JIA.

## Background

Juvenile idiopathic arthritis (JIA) is the most common inflammatory rheumatic disease in children and is an autoimmune disease of unknown origin. Apart from cells of the innate immune system like neutrophils and monocytes, which trigger inflammation, T cells play a dominant role in the inflammatory reaction of the joint. Recent investigations indicate an accumulation of highly inflammatory CD4^+^CD161^+^ cells in the joints [1–3]. Although the functional relevance of CD161 ligation on T cell function is less clear, CD161 expression is a useful indicator of inflammatory T cells. They belong to either Th1, Th17 or Th1/Th17, so called non-classical Th1, cells and their proportions in synovial fluid (SF) correlate positively with parameters of disease activity [1, 4, 5]. The inflammatory potential of these effector T cells within the joint is controlled by regulatory T cells (Tregs). The control, however, is inefficient despite the high presence of Tregs in arthritic joints. Tregs typically are not pro-inflammatory, but recent reports showed that some Tregs also may share functional capabilities with conventional T cells, like production of inflammatory cytokines in the context of autoimmunity or chronic inflammation [6–8]. These Tregs are part of the CD161 population and also enriched in joints of JIA and rheumatoid arthritis (RA) patients [2, 9].

The cAMP response element modulator (CREM) α binds to promoters of genes with cAMP response elements (CRE) and regulates transcription via a chromatin-dependent mechanism. Under physiological conditions, the production of CREM is tightly regulated and involves the differential use of alternate promoters and splicing processes, resulting in cell- and tissue-specific expression patterns [10, 11]. Quite interesting is that in T cells of SLE patients CREMα mRNA and protein expression is increased and this significantly alters the expression of various T lymphocyte-specific target genes, including IL-2 and IL-17 family cytokines [12–15]. Notably, the observed effects of CREMα on IL-2 and IL-17a cytokine production in humans are also observed in transgenic mice with T cell-specific CREMα overexpression [16]. These mice have decreased IL-2 and increased IL-17a levels and are more prone to develop signs of autoimmunity (including lymphadenopathy and higher autoantibody titers against double-stranded DNA) when an additional genetic deletion of the *cd95* gene (Fas) is present [16, 17]. Beyond its role in SLE CREMα also contributes to T cell dysregulations in asthma, LPS-induced lung injury, colitis, and EAE [18–21]. Although it is known that T cells contribute to pathogenesis in JIA, the role of CREM here has not been addressed so far.The aim of this study was to evaluate the role CREM expressing T cells in oligoarticular JIA. Our findings indicate that beyond its role in SLE CREMα also contributes to T cell pathophysiology in oligoarticular JIA by modulating inflammatory and regulatory T cells.

## Methods

### Flow cytometry

For surface staining, single cell suspensions were stained with anti-CD3 (UCHT1), anti-CD4 (RPA-T4), anti-CD161 (HP-3G10) antibodies (all from eBioscience, Germany). To analyze Foxp3 and CREM expression, cells were fixed and permeabilized with a FOXP3 staining buffer set (eBioscience, Germany) following the manufacturer’s instructions and stained with anti-Foxp3 (PCH101) antibodies (eBioscience, Germany), monoclonal anti-CREM (Abcam, Great Britain) or IgG isotype control antibodies for 30 min. Monoclonal anti-CREM antibodies and IgG isotype control antibodies were labeled with Alexa Fluor Antibody Labeling Kits (Thermo Fisher Scientific, USA) according to manufactures instructions. For measurement of intracellular cytokines, cells were treated with propidium iodide (P/I) and GolgiPlug (BD Bisciences, Germany) for 5 h and fixed and permeabilized with FoxP3 staining buffer set (eBioscience, Germany) following the manufacturers’ instructions. Intracellular cytokines were stained with anti-IFN-γ (4S.B3) APC and anti-IL-17 PE (64DEC17) (both eBioscience, Germany) antibodies.

### Patients and healthy donors

All patients were diagnosed as having oligoarticular JIA and were receiving nonsteroidal anti-inflammatory drugs before therapeutic aspiration of SF and administration of corticosteroids. JIA patients were diagnosed according to internationally agreed criteria.Cells were pelleted by centrifugation and supernatants were individually stored at − 20 °C, with this more than twenty different SFs and HC sera were collected and are included in different experiment in this study. Ethical approval for all experiments was obtained from the local ethics committee. All patients provided fully informed consent or age-appropriate assent where applicable. Sera from healthy controls (HC) were obtained from peripheral blood. For co-incubation wit HC Sera and SF, cells from healthy donors were isolated from buffy coats provided by the local blood bank, Transfusionsmedizin, UniversitätsklinikumAachen, Germany).

### Cell isolation

Human mononuclear cells from patients with JIA were isolated onto a Ficoll (PAN Biotech, Germany) gradient either from peripheral blood (PB) or synovial fluid (SF). Erythrocytes were lysed and cells were washed twice. Peripheral blood mononuclear cells (PBMC) were isolated from healthy donors by the same procedure.

### Cell culture

PBMCs from healthy donors were incubated with 10% allogenic SF or serum from allogenic healthy controls (HC) in RPMI (Gibco, Germany) with 10% FCS (Biochrom, Germany). When indicated, cells were stimulated with plate-bound anti-CD3 and anti-CD28 antibodies (both at 3 μg/ml; BD Bioscience, Germany) in individual wells of 96-well round-bottom microtiter plates. To knock-down CREM expression, PBMCs and SFMCs were transfected with 5 nM CREM-specific siRNA or irrelevant control siRNA (Origene, USA) using the Amaxa transfection system (Lonza, Switzerland). After four hours cells were transferred in fresh media and either left unstimulated and analyzed after 24 h or stimulated and anyalzed as indicated.

### RNA isolation, complementary DNA (cDNA) synthesis, and quantitative real-time polymerase chain reaction (PCR)

Total RNA was extracted from cells using an RNeasy Mini Kit (Qiagen, Germany) and transcribed to cDNA using a First Strand cDNA Synthesis Kit (Thermo Fisher Scintific, USA) according to the manufacturer’s instructions. Standard quantitative real-time PCR was carried out on a TaqMan 7900 (Applied Biosystems, USA) using the DNA intercalating dye SYBR Green.

### Ovalbumin (OVA)-induced arthritis model

OVA-induced arthritis was induced in mice, as described previously [22]. Briefly, 2 × 10^6^ OVA-specific CD4^+^CD25^−^ T cells from either wild-type (WT) or CREM^−/−^ OT-II mice were injected intraperitoneally into C57BL/6 RAG^−/−^ mice. Two independent experiments were performed and overall 6 mice per group were analyzed. One day later recipients were immunized with 100 μg cationized OVA (Sigma-Aldrich, Germany) in PBS at the base of the tail. On day 7 mice were rechallenged with 60 μg cationized OVA injected intra-articularly into the left knee joint. Knee swelling was assessed using calipers at definite time points as the difference between the right (arthritic) before and after OVA-injection.

### Mice

Experiments were performed withage-matched RAG^−/−^, WT OT-II and CREM^−/−^ OT-II mice (all C57BL/6). CREM^−/−^ animals were originally cloned and provided by Prof. G. Schütz (Deutsches Krebsforschungszentrum, Heidelberg, Germany) [23]. CREM^−/−^ OT-II mice were generated by crossing CREM^−/−^ mice with OT-II mice. All mice were bred in our animal facility and kept under standardized conditions. The study was approved by the regional government authorities and animal procedures were performed according to German legislation for animal protection.

### Histology

Knee joints from mice were fixed in 4% neutral buffered formalin solution for 24 h. Afterwards they were placed in an EDTA-decalcifying solution (20% EDTA) for 20 days, dehydrated, and embedded in paraffin blocks. Sections were cut along a longitudinal axis at 6 μm and stained with hematoxylin and eosin. Hematoxylin and eosin stained slides were evaluated and scored blindly for exudates, granulocyte infiltration, hyperplasia, fibroblast proliferation/mononuclear cell infiltration, periarticular mononuclear cell infiltration (each scoring 0–3), bone/cartilage destruction (scoring 0–4), and an additional score of 1 for visible fibrin deposition and periarticular granulocyte infiltration, resulting in a maximum score of 21.

### Statistical analysis

All data are presented as mean ± standard error (SEM). Differences between two groups were evaluated using two-tailed unpaired or paired (if indicated), Student’s t-test if data were normally distributed. Otherwise, a non-parametric Mann-Whitney test or Wilcoxon matched-pairs signed rank test were performed. All statistical analysis and subsequent graphics generation were performed using GraphPad Prism version 7.0 (GraphPad Software, USA). A *p*-value < 0.05 was considered to be statistically significant.

## Results

### CREMα is overexpressed in synovial fluid T cells of juvenile arthritis patients

**Fig. 1:**
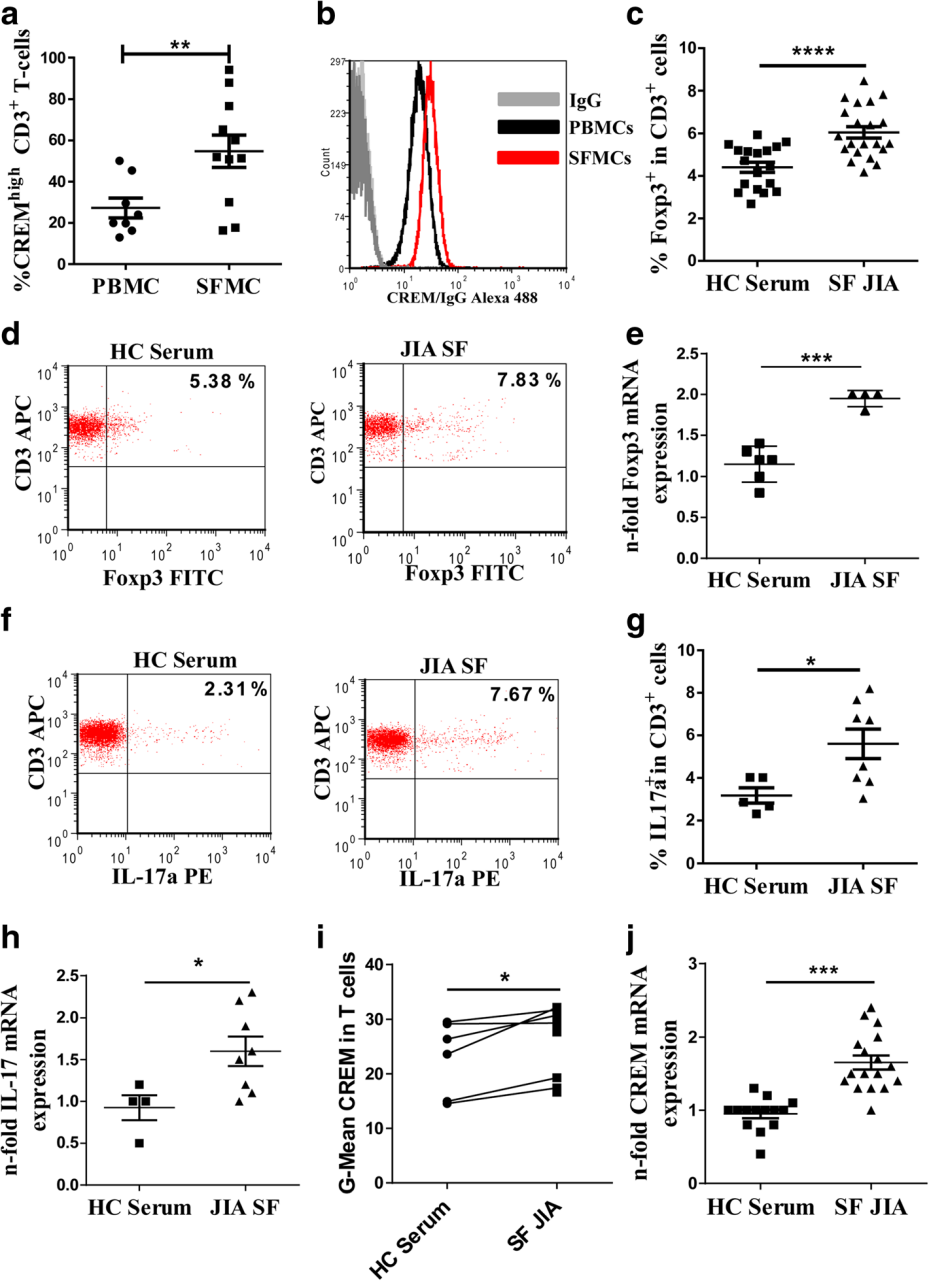
CREM is overexpressed in JIA T cells. **a** Percentages of CREM high expressing T cells of 8 healthy control PBMCs and of 11 oligoarticular JIA SFMCs as assessed by flow cytometry. **b** Representative histogram showing mean fluorescent intensity of CREM expression in CD3^+^ T cells. **c-j** HC PBMCs were treated with 10% SF or HC Serum in RPMI for 24 h. **c** Percentages of Foxp3^+^ cells within CD3^+^ T cells. **d** Representative dot plot of Foxp3 expressing T cells. **e** Foxp3 mRNA expression. **f** Representative dot plot of IL-17^+^ expressing T cells after restimulation with P/I in the presence of Brefeldin A. **g** Percentages of IL-17^+^ cells within CD3^+^ T cells after restimulation with P/I in the presence of Brefeldin A. **h** IL-17 mRNA expression. **i** Geometric (G)-Mean of CREM expression, paired, two tailed t-test. **j** CREM mRNA expression, two-tailed Mann Whitney test. Symbols present individual patients, SFs or HC sera and horizontal bars show SEM. A two-tailed unpaired t-test was used to calculate *p*-values in **a, b, e, g** and **h**

T cells from SLE patients have previously been shown to display enhanced CREMα levels, thus pointing to the relevance of CREMα in human disease. Based on these findings we asked if expression of CREMα is also upregulated during another autoimmune disease in which T cells are involved in pathogenesis. We therefore investigated expression of CREMα in synovial fluid T cells from JIA patients. As shown in Fig. 1, percentages of CREM^high^ cells within CD3^+^ T cells were indeed more abundant in the synovial fluid of JIA patients than in the peripheral blood of healthy controls (Fig. 1a and b).

### Incubation of control PBMCs with synovial fluid upregulates FoxP3 and IL-17 expression and involves upregulation of CREM

Synovial fluid of JIA patients contains high amounts of FoxP3^+^ and IL-17^+^ cells [24, 25]. Hence, we asked whether SF of JIA patients contains factors responsible for differentiation of these type of immune cells and whether this went along with increased expression of CREM. Thus we mimicked the inflammatory setting in the joint in vitro by incubating PBMCs from healthy controls with 10% allogenic synovial fluid from individual patients for 24 h. As a control we incubated PBMCs from the same healthy controls with 10% allogenic serum from individual healthy controls for 24 h. We observed increased percentages of FoxP3 positive cells in particular (Fig. 1c and d) and an increase in FoxP3 mRNA in general (Fig. 1e) when PBMC were incubated in the presence of synovial fluid for 24 h. In addition, when restimulated with P/I in the presence of Brefeldin A, percentages of IL-17^+^ T cells (Fig. 1f and g) and IL-17 mRNA (Fig. 1h) were also increased in SF-stimulated PBMC. Moreover, incubation with SF also upregulated CREM mRNA expression in PBMC (Fig. 1j) and CREM protein expression specifically in T cells (Fig. 1i). Hence, incubation of control PBMCs with SF not only upregulates FoxP3 and IL17 expression but also CREM expression in T cells. Therefore, we hypothesize that soluble factors within the synovial fluid induce CREM transcription in SF T cells which leads to enhanced expression of CREM in SF T cells.

### SF-induced expression of IFN-γ, IL-17, and FoxP3 in T cells can be reversed by CREM knock down with CREM-specific siRNA

**Fig. 2:**
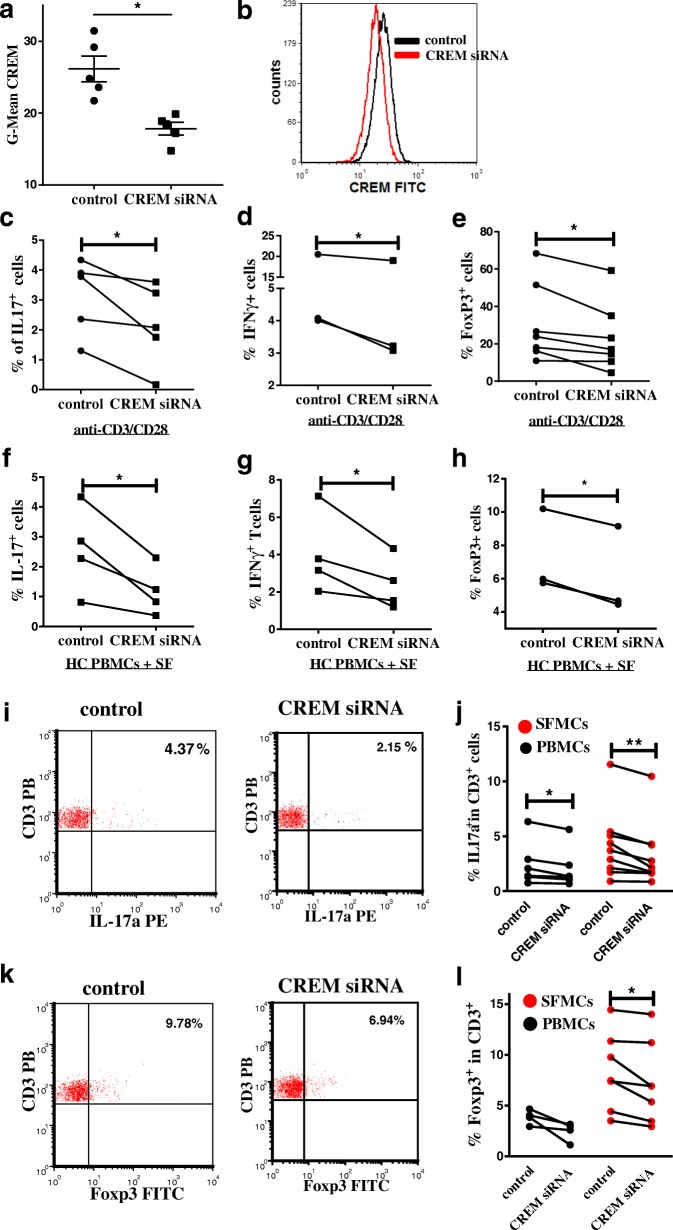
CREM contributes to T cell dysregulations in JIA. **a** Healthy control PBMCs were transfected with control siRNA or with CREM siRNA and G-Mean of CREM expression was analyzed by flow cytometry, two-tailed paired t-test. **b** Representative histogram of CREM expression after transfection with control siRNA (black) or CREM siRNA (red). **c-e** Healthy control PBMCs were transfected with control siRNA or with CREM siRNA and stimulated with anti-CD3 and anti CD28 antibodies for 3 days, symbols present individual healthy controls incubated with different allogenic HC sera or SFs, two-tailed paired t-tests**. c** Percentages of IL-17^+^ cells within CD3^+^ T cells after restimulation with P/I in the presence of Brefeldin A. **d** Percentages of IFN-γ^+^ cells within CD3^+^ T cells after restimulation with P/I in the presence of Brefeldin. **e** Percentages of Foxp3^+^ cells within CD3^+^ T cells. F-H) Healthy control PBMCs were transfected with control siRNA or with CREM siRNA and incubated with 10% SF in RPMI for 24 h, two-tailed, paired t-tests were used to calculate *p*-values. **f** Percentages of IL-17^+^ cells within CD3^+^ T cells **g** Percentages of IFN-γ^+^ cells within CD3^+^ T cells. **h** Percentages of Foxp3^+^ cells within CD3^+^ T cells after restimulation with P/I in the presence of Brefeldin A. **i-l** PBMCs and SFMCs from JIA patients were transfected with control siRNA or with CREM siRNA and **i-h**) percentages of IL-17^+^ cells after restimulation with P/I in the presence of Brefeldin A, Wilcoxon matched-pairs signed rank test was used to calculate p-values and of **k-l**) Foxp3^+^ cells were assessed by flow cytometry after 24 h, two-tailed paired t-tests were used to calculate p-values

We next asked if expression of CREM directly influences activation of T cells and therefore analyzed T cell activation in the absence of CREM. We isolated CD4^+^CD25^−^ cells from healthy donors, transfected them with CREM-specific siRNA or control siRNA. Flow cytometric analysis revealed that siRNA knockdown results in reduced protein expression of CREM (Fig. 2a and b). We next incubated the cells with anti-CD3/CD28 antibodies. Our analysis showed that percentages of IL-17a^+^, IFN-γ^+^ and FoxP3^+^ cells were significantly reduced in CREM siRNA transfected cells (Fig. 2c, d and e). To further analyze if CREM is required for IL-17a and FoxP3 expression under the inflammatory environment we stimulated CD4^+^ T cells with knocked down CREM by incubation in SF from JIA patients. Stimulation of CD4^+^ T cells with healthy control serum served as control. Similar to anti-CD3/CD28 antibody mediated stimulation of CD4^+^ T cells, also the SF-induced expression of IFN-γ, IL-17a and FoxP3 could be reversed in CD4^+^ T cells by expression of CREM-specific siRNA (Fig. 2f, g, and h). To establish the involvement of CREMα in the regulation of IL-17a, FoxP3 and IFN-γ expression in JIA we transfected ex vivo isolated SFMC with either unrelated control siRNA or siRNA directed against CREM. SFMC transfected with CREM-specific siRNA expressed significantly lower percentages of FoxP3^+^ and IL17a^+^ cells (Fig. 2i-l). Hence, these experiments provide evidence for the involvement of CREMα in the regulation of IL-17 and FoxP3 expression in juvenile arthritic joints.

### CREM regulates inflammatory CD161^+^ T cells and determines the outcome in inflammatory arthritis

**Fig. 3:**
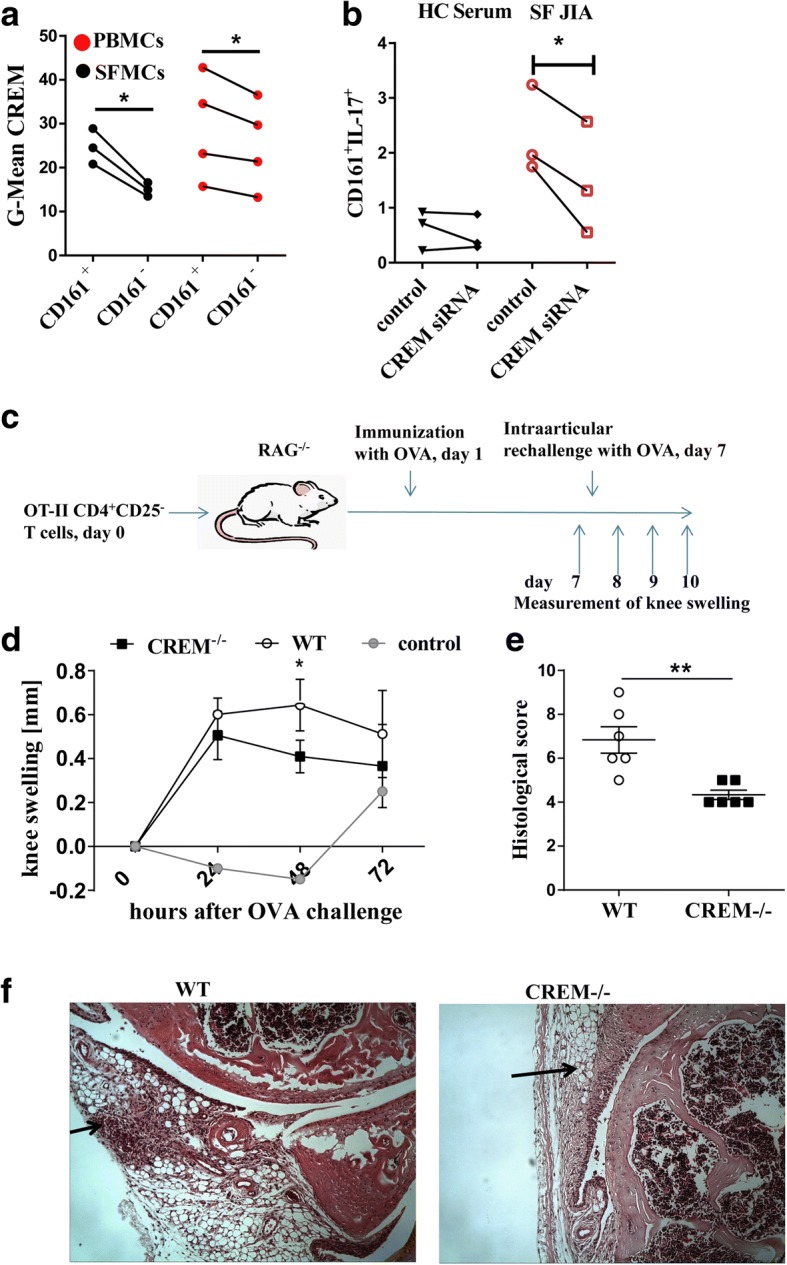
CREM regulates T cell inflammation in arthritis. **a** Geometric (G)-Mean of CREM expression in PBMCs and SFMCs from JIA patients, two-tailed paired t-tests were used to calculate p-values. **b** PBMCs and SFMCs from JIA patients were transfected with control siRNA or with CREM siRNA and cultivated in RPMI for 24 h and percentages of CD161^+^IL-17^+^ in CD3^+^ T cells after restimulation with P/I in the presence of Brefeldin were determined, two-tailed paired t-tests were used to calculate p-values. **c** Graphic showing T cell mediated arthritis model. **d** Knee swelling of RAG^−/−^ mice after adoptive transfer of OT-II cells either from WT or from CREM^−/−^ mice, immunization with OVA and subsequent intraarticular injection of cationic ovalbumin. **e** Histological scores of arthritis-induced knee joints, Mann Whitney test were used to calculate *p*-values. **f** Hematoxylin and eosin staining of knee joint sections (arrow on the loft shows cellular infiltration, arrow on the right shows thickening of the synovial membrane)

Among CD4^+^ cells the subset of CD161^+^ T cells are the most important in maintaining the inflammatory process and exactly this cell population is increased in the SF of JIA patients [1, 5]. We thus analyzed the role of CREMα within this population of CD4^+^ cells. We found enhanced expression of CREMα in CD161^+^CD4^+^ PBMC and of SFMC from JIA patients compared to their CD161^−^ counterparts (Fig. 3a). Treatment of HC PBMCs with SF upregulated CD161^+^CD4^+^IL-17^+^ cells in the presence of CREM, while knock down of CREM inhibited the SF induced expression of this inflammatory subset (Fig. 3b). Unfortunately, in contrast to humans, mice do not express CD161 in cytokine-producing T cells and therefore we could not validate these data in vivo. Nevertheless, we performed a T cell dependent arthritis model in mice to analyze how CREM signaling in T cells influences the fate of an inflammatory arthritis.

To this end, we transferred CD4^+^CD25^−^ T cells from either OTII-CREM^−/−^ or control OTII-wild-type (WT) mice into RAG^−/−^ mice and immunized the mice with OVA to expand the T cells (Fig. 3c). On day 7 we induced arthritis by injecting cationic OVA-peptide into the right knee. As seen in Fig. 3d, transfer of CREM^−/−^ T cells resulted in a faster remission of arthritis and significantly lower histological scores for inflammation and tissue destruction (Fig. 3d-f).

## Discussion

For the first time we provide evidence that CREM plays a role in T cell dysregulation in oligoarticular JIA patients. Our conclusion is based on several levels of evidence. First, we observed enhanced expression of CREM in SF T cells from JIA patients. Enhanced expression of CREM could also be induced after ex vivo culture of PBMCs from healthy donors with SF from JIA patients. CREM expression is also enhanced in SLE T cells and as well as SF Sera from SLE patients also induces CREM expression [26]. However, expression of CREM is regulated by complex mechanism and by at least two different promoter regions that are differentially activated in SLE and normal T cells [10]. Further studies will show which promoter regions are activated by SF. Second, we found enhanced expression of CREM in CD4^+^CD161^+^ cells, which are known producers of inflammatory cytokines. Third, incubation with SF induced expression of IFN-γ, IL-17 and FoxP3 in T cells, which could be reversed by knock down of CREM. Finally deficiency of CREM in T cells ameliorated OVA induced arthritis in vivo.

There are some limitations to our study. While our data suggest that CREM directly regulates CD4^+^CD161^+^ T cells in human JIA, we cannot fully transfer this observation to our in vivo arthritis model as the murine analog of CD161 has not yet been identified. Furthermore, we could only analyze a small number of patients and further work is required to confirm our data.

Regarding the pathophysiology of JIA, the inflammatory reaction within the joint is initiated by cells of the innate immune system like neutrophils and macrophages, but cells of the adaptive immune system like B cells and T cells play a dominant role in perpetuating the disease. Pathogenic T cells within the joint display a mixed Th17/Th1 phenotype characterized by the production of IL-17 as well as IFN-γ and expression of both lineage transcription factors T-bet and RORyT [2]. Furthermore, in humans, these cells display a high expression of CD161, low expression of the TCR ζ chain (CD247) and low expression of IL-2 as well as low response to stimulation with IL-2 [27]. In addition, the TCR ζ chain has been established recently as an independent risk factor for JIA in linkage analysis studies [28]. The same is true for the IL2- receptor [29]. Interestingly, despite abundance of pathogenic T cells within the inflamed joint, the expression of detectable IL-2 within the joint is negligible [30]. This could be the result of decreased expression of IL-2 by the Th17 cells [27] or of consumption by the abundant regulatory T cells, which are dependent on IL-2. CREMα has been shown before to downregulate the TCR ζ chain and the IL-2 expression in SLE while enhancing secretion of IL-17 and IL-21 [12, 13, 16, 20, 31] and as shown here also dysregulates T cell responses in JIA as shown by our siRNA and in vivo studies. We therefore suggest that CREM regulates T cells in JIA by several mechanisms, which similarly to consequences of CREM overexpression in SLE contributes to an aberrant cytokine-expression profile and an enhanced occurrence of Th17 cells. How exactly CREM is activated in the synovium remains to be elucidated.

Recent studies underline the importance of a balance between inflammatory T cells and Tregs within inflamed joints [32] and CREM mediated mechanism might have potential as a therapeutic strategy for Th17-driven autoimmune diseases. It is therefore noticeable that genetic or pharmacologic inhibition of calcium/calmodulin-dependent protein kinase IV (CaMK4) reduced Il-17 transcription through decreased activation of CREMα [21]. Furthermore a recent clinical trial demonstrated the safety and efficacy of low-dose IL-2 treatment on SLE [33]. This shows that there are already advances to re-establish CREM-mediated dysregulations in T cells in autoimmune diseases and our study suggests that it would be valuable to further analyze the therapeutic potential of these mechanisms in JIA as well.

## Conclusion

T cell dysregulations critically contribute to ongoing inflammation in juvenile arthritis joints. By identifying CREM as a transcriptional activator that contributes to increased occurrence of inflammatory effector T cells within the joints; our study puts CREMα in a central role within JIA and makes it a possible attractive target for pharmacological intervention.

## Abbreviations

CREM: cAMP responsive element modulator
HC: Healthy control
JIA: Juvenile idiopathic arthritis,
OVA: Ovalbumin
PB: Peripheral blood
PBMC: Peripheral blood mononuclear cells
SF: Synovial fluid
